# The left-lateralisation of citrate synthase activity in the anterior cingulate cortex of male violent suicide victims

**DOI:** 10.1007/s00406-022-01509-2

**Published:** 2022-11-09

**Authors:** Karol Karnecki, Julian Świerczyński, Johann Steiner, Marta Krzyżanowska, Michał Kaliszan, Tomasz Gos

**Affiliations:** 1 Department of Forensic Medicine, Medical University of Gdańsk, Ul. Dębowa 23, 80-204 Gdańsk, Poland; 2 Department of Biochemistry, Medical University of Gdańsk, Gdańsk, Poland; 3 Department of Psychiatry, Otto von Guericke University, Magdeburg, Germany

**Keywords:** Postmortem, Suicide, Anterior cingulate cortex, Citrate synthase

## Abstract

The anterior cingulate cortex (AC) as a part of prefrontal cortex plays a crucial role in behavioural regulation, which is profoundly disturbed in suicide. Citrate synthase (CS) is a key enzyme of tricarboxylic acid cycle fundamental for brain energetics and neurotransmitter synthesis, which are deteriorated in suicidal behaviour. However, CS activity has not been yet studied in brain structures of suicide victims. CS activity assay was performed bilaterally on frozen samples of the rostral part of the AC of 24 violent suicide completers (21 males and 3 females) with unknown psychiatric diagnosis and 24 non-suicidal controls (20 males and 4 females). Compared to controls, suicide victims revealed decreased CS activity in the right AC, however, insignificant. Further statistical analysis of laterality index revealed the left-lateralisation of CS activity in the AC in male suicides compared to male controls (*U*-test *P* = 0.0003, corrected for multiple comparisons). The results were not confounded by postmortem interval, blood alcohol concentration, age, and brain weight. Our findings suggest that disturbed CS activity in the AC plays a role in suicide pathogenesis and correspond with our previous morphological and molecular studies of prefrontal regions in suicide.

**Supplementary Information:**

The online version contains supplementary material available at 10.1007/s00406-022-01509-2.

## Introduction

**Fig. 1:**
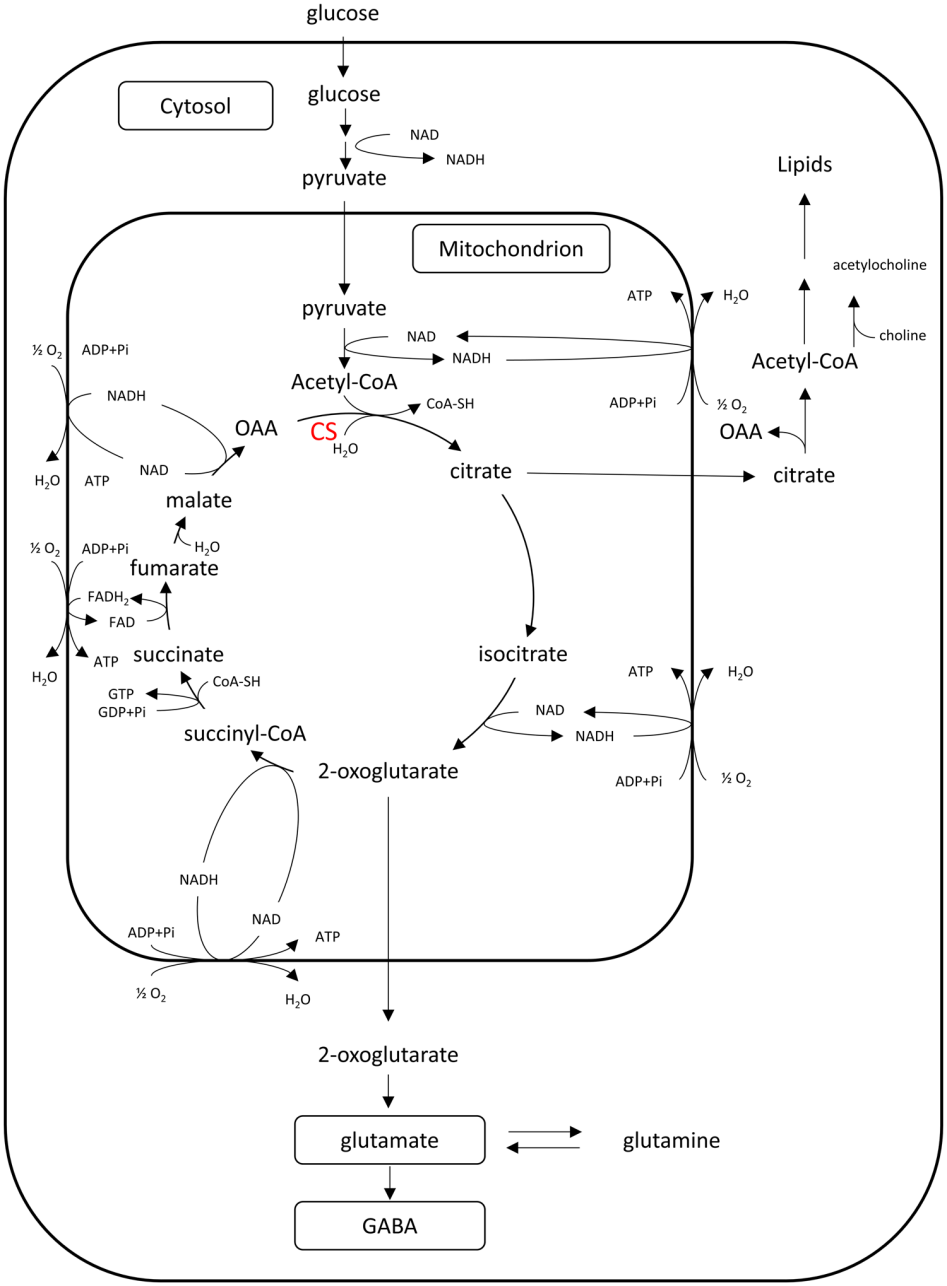
The key metabolic roles of citrate synthase in the brain: the involvement in brain energetics (i.e. in the biosynthesis of ATP), and in the biosynthesis of neurotransmitters and lipids. NADH formed in reactions catalyzed by NAD-dependent dehydrogenases of tricarboxylic acid cycle (isocitrate dehydrogenase, 2-oxoglutarate dehydrogenase, malate dehydrogenase) and pyruvate dehydrogenase is oxidized by respiratory chain located in the inner mitochondrial membrane. This process is coupled with ATP synthesis (from ADP + Pi). Similarly, FADH_2_ formed in the reaction catalyzed by succinate dehydrogenase is oxidized by respiratory chain located in inner mitochondrial membrane and this process is also coupled with ATP synthesis. The first step in tricarboxylic acid cycle is the biosynthesis of citrate by citrate synthase, which is a key enzyme in this cycle. *CS* citrate synthase, *OAA* oxaloacetate, *CoA* Coenzyme A, *GABA* gamma-aminobutyric acid

Citrate synthase (EC 4.1.3.7) is localized within cells in the mitochondrial matrix and catalyses the condensation of oxaloacetate and the acetyl group of acetyl coenzyme-A (acetyl CoA), which yields citrate and CoA [1, 2]. This is a key reaction of the tricarboxylic acid (TCA) cycle (Krebs cycle), which plays a fundamental role in glucose oxidation, and thus in brain energy metabolism [3, 4], disturbed in mental disorders [5]. Besides energy production, the main aspects of CS activity in the brain are neurotransmitter synthesis and lipids metabolism [6] (Fig. 1).

The TCA cycle in astrocytes with CS as a key component plays a fundamental role in the replenishing of both glutamate and GABA transmitter pools in neurons, i.e. the most important excitatory and inhibitory brain neurotransmitters, respectively [6–9]. Citrate released in large amounts from astrocytes may act as a chelator of extracellular divalent cations, and thus as a modulator of receptor function. In particular, the multifaceted activity of glutamatergic N-methyl-D-aspartate receptors (NMDARs) may be upregulated by this chelating action on Mg^2+^ [6]. The increased activity of prefrontal NMDARs was suggested in depressed suicide victims [10] and citrate increase was previously shown in the cerebrospinal fluid of depressed patients, which was normalised by antidepressants [11]. Besides the above-mentioned chelating and metabolic roles, citrate serves in the brain as a substrate for the biosynthesis of another excitatory neurotransmitter—acetylcholine [12, 13]. Therefore, experimental studies have suggested for decades an involvement of CS in cortical activity [14, 15].

Limbic regions of prefrontal cortex (PFC), i.e. the anterior cingulate cortex (AC) and the orbitofrontal cortex play a key regulatory role in emotionally influenced behaviour and their abnormalities are consistently reported in depression and suicide [10, 16–18]. Experimental research on animal models of depression suggests an involvement of disturbed prefrontal CS activity in the pathogenesis of this mental disorder [19, 20], which is frequently comorbid with suicide [10, 16]. Different antidepressant and/or antipsychotic regimens modulated (predominantly increased) CS activity in the PFC of experimental animals, which supports this concept [1, 2, 21–24].

CS activity in human frontal cortex samples stored at − 80 °C was not affected by postmortem interval (range: 2.5 to 26 h), storage time (11.8 to 104.1 months), age of the donor (2 days to 80 years), and agonal state (Glasgow Coma Scale score: 3 to 15) [25]. No differences in this activity were found postmortem in the PFC in small cohorts of Alzheimer disease [26] and schizophrenia patients [27]. However, no studies of CS activity in brain structures have yet been reported in suicide victims.

Therefore, in the present study, we hypothesized a disturbed CS activity in the AC of suicide completers regardless of their underlying psychiatric diagnosis (i.e. independent of psychiatric comorbidity) and tested this hypothesis by the assessment of CS activity in forensic postmortem material. We aimed at both basic research on the neurobiology of suicide and the informative comparison between our current biochemical and previous morphological and molecular evaluation of the disturbed PFC function in suicide victims [16–18].

## Materials and methods

### Human brain tissue

Prefrontal parts of both hemispheres of 24 suicide victims (21 males/3 females) with unknown data both on psychiatric comorbidity and on possible psychotropic medication preceding death (typical for most of suicide cases autopsied in the Department of Forensic Medicine at the Medical University of Gdańsk) and 24 (20 males/4 females) controls were obtained during routine forensic autopsies in accordance with existing EU law regulations [17, 18]. The study has been approved by the local ethics committee of the Medical University of Gdańsk as performed in accordance with the ethical standards laid down in the Declaration of Helsinki of 1989.

Detailed diagnostic and demographic data of investigated cases are present in the Supplementary Table. The suicide cohort included violent suicides, which prevail in our autopsy material. Control cases of natural manner of death were more numerous than those of unnatural manner (20 and 4, respectively). Only sudden death cases were investigated in suicide and control cohorts. All brains were free of gross neuropathology suggestive of vascular, traumatic, inflammatory, neoplastic and neurodegenerative processes. Macroscopic evaluation of brains was confirmed by histopathological investigation in cases, where the cause of death was unclear at autopsy and the routine histopathological evaluation of internal organs was necessary for the forensic diagnosis (i.e. in 20 control cases of natural manner of death). Neither chronic nor acute pathological processes were observed microscopically in these cases in neocortical areas and other brain regions in hematoxylin–eosin stained sections. Among others, neuronal necrosis as a consequence of protracted antemortem hypoxia was excluded by histopathological investigation. Neurodegenerative changes such as amyloid plaques, perivascular amyloid deposits and neurofibrillary tangles were not observed microscopically in the AgNOR silver staining in prefrontal regions in those cases, which were included in cohorts investigated previously [17]. Blood was tested for the presence of ethanol at each autopsy. The majority of investigated cases (12 suicide victims and 15 controls) revealed the blood alcohol concentration (BAC) below the limit of quantification (LOQ), i.e. < 0.2 g/l according to internationally accepted analytical guidelines. The remaining 12 suicide victims and 9 controls revealed BAC in the range of 0.24–2.8 g/l (the highest value in one of hanging cases) and 0.3–2.8 g/l (the highest value in the victim of transport accident), respectively.

Prefrontal parts of the brains were separated at forensic autopsies from both hemispheres by coronal sections at the level of temporal poles. Immediately after the separation, cortical samples for CS assays were isolated bilaterally from the rostral (pregenual) part of the AC located closely to the genu of corpus callosum. Each sample was approximately 10 mm in length, 5 mm in width, and 2–3 mm thick, i.e. cortical samples were isolated by an experienced forensic pathologist (KK) under visual control at the clearly visible border with the subcortical white matter (thus each cortical sample contained all cortical layers). Immediately after the isolation, cortical samples were transferred to the deep-freezing refrigerator and stored at − 80 °C. After sampling procedure the remaining prefrontal parts were preserved for the morphological and molecular investigations, which were presented previously [17, 18].

### Citrate synthase activity assay

Approximately 0.1 g AC sample was placed in 3 ml of 20 mmol/L Tris chloride buffer pH 7.8 containing 0.2% Triton X-100. The tissue was thawed, minced finely with scissors, homogenized manually with a Teflon-pestle homogenizer (small size), and centrifuged at 30,000 g for 20 min. The resulting supernatant was decanted, and the pellet was resuspended in 2 mL of isolation medium, rehomogenised and centrifuged as above. The supernatant was combined with that obtained after the first centrifugation step and used for enzyme assay.

CS activity was measured by following the formation of 5-thio-2-nitrobenzoic acid (TNB) during the reaction: CoA-SH + 5,5’dithio-bis-2-nitro-benzoic acid (DTNB) → CoA-S–S-TNB (yellow product), coupled with the reaction catalyzed by citrate synthase: oxaloacetate + acetyl-CoA + H_2_O → citrate + CoA-SH, as described previously [28].

Briefly, the assay medium (final volume 1 mL) contained: 100 mmol/L Tris–HCL pH 8.1, 0.1 mmol/L DTNB, 0.5 mmol/L acetyl CoA, and 0.5 mmol/L oxaloacetate (OAA). The reaction was started by adding OAA. The assay was performed in duplicate at 37 °C. The yellow product CoA-S–S-TNB was quantified by measuring absorbance at 412 nm (molar absorption coefficient 13.6 × mM^−1^ × cm^−1^) using a Beckman DU68 spectrophotometer (Beckman Instruments, Fullerton, CA, USA). Absorbance changes were linear against both time and enzyme concentration. Enzyme activity was expressed as nmol × min^−1^ × mg^−1^ protein. Protein assays were performed according to the Peterson’s method [29].

### Data analysis

Statistical analyses were performed with the data analysis software system STATISTICA version 10 (StatSoft®, Inc. 2011, www.statsoft.com). As normal distribution was not given for analysed data (i.e. significant values of Kolmogorov–Smirnov and Lilliefors tests were obtained), non-parametric statistical procedures were used in hierarchic mode.

First, STATISTICA generalized linear/nonlinear models (GLZ) module containing general custom designs (GCD) procedure was applied as an omnibus method to analyse associations between dependent variable (i.e. CS activity in the AC bilaterally) and independent categorical variables (i.e. suicidal/control group, brain hemisphere, and sex as the categorical confounding variable). The results of the GCD analysis were reported automatically including the Wald statistic value, degrees of freedom, and the respective *P* value.

Furthermore, the laterality index of CS activity in the AC (100 × [left − right]/[left + right]) was calculated in each case to compare the lateralisation effect between study groups. Age, postmortem interval, brain weight and BAC (values below LOQ were accounted null values in statistical analysis) were considered as numerical confounding variables. Therefore, the subsequent GCD procedure was applied to analyse associations between these variables and dependent variables, i.e. CS activity bilaterally and laterality index. Supplementary to GCD analyses, Spearman’s correlation coefficients were calculated to determine the impact of these variables which might confound the dependent variables.

Following the GCD analysis, unadjusted two-way *post hoc* comparisons with Mann–Whitney *U*-test and the *χ*^*2*^-test were used to detect possible differences between the studied groups with respect to the variables mentioned above (i.e. CS activity, laterality index, and confounders). All statistical tests were two-tailed. In general, *P* values of < 0.05 were accepted as statistically significant.

Kruskal–Wallis analysis of the variance of ranks (*H*-test) with subsequent *U*-tests were performed for the evaluation of differences in CS activity and laterality index related to sex between suicides and controls; in this procedure *U*-test *P*-values were adjusted to multiple comparisons according to the Bonferroni correction. The differences in investigated parameters related to BAC levels were analysed in a comparable manner (i.e. in cases with BAC values higher than LOQ versus remaining cases).

## Results

### The analysis of CS activity

Cumulative analysis of results from the AC bilaterally (i.e. 96 suicidal and 96 control values) by the GCD procedure suggested differences in CS activity associated with sex (Wald statistic = 19.64, df = 1, *P* = 0.000009).

Further analyses by *U*-tests revealed an increased laterality index in suicides compared to controls due to the left-lateralised CS activity in the AC in the former study group (*U*-test *P* = 0.00009), which was driven mainly by male subjects (see next paragraph). However, the inter-group difference in CS activity observed in the right AC (i.e. a decrease in suicides compared to controls) was insignificant, which could be related to the accentuated variability of results (see Tables 1, 2, 3 and Supplementary Table).

**Table 1:** Intergroup comparisons of dependent variables, i.e. citrate synthase activity (CS, nmol × min−1 × mg protein−1) in the anterior cingulate cortex (AC) bilaterally, and the laterality index of CS activity (LI, 100 × [left − right]/[left + right])

		CS, AC left	CS, AC right	LI, all cases	LI, males
Suicides: median (q1, q3, *n*)		177.59 (147.05, 198.73, 24)	158.90 (139.39, 185.00, 24)	5.08 (0.35, 6.34, 24)	5.16 (0.69, 6.27, 21)
Controls: median (q1, q3, *n*)		175.72 (132.70, 198.00, 24)	175.50 (134.66, 190.93, 24)	− 1.74 (− 4.55, 0.79, 24)	− 2.73 (− 4.55, − 0.43, 20)
Statistics:					
Test		U	U	U	U
Characteristic value		Z = 0.577	Z = − 0.825	Z = 3.763	Z = 3.625
*P* value		0.560	0.407	**0.00009**	**0.0003**

**Table 2:** Intergroup comparisons of confounding variables

		Age [yrs.]	PMI [hrs.]	BW [g]	BAC [g/l]
Suicides: median (q, q3)		48.5 (26, 60)	36 (24, 72)	1470 (1375, 1525)	0.12 (0.00, 1.31)
Controls: median (q1, q3)		53 (42, 59.5)	48 (24, 84)	1345 (1275, 1478)	0.00 (0.00, 1.06)
Statistics:					
Test		U	U	U	U
Characteristic value		Z = − 1.041	Z = − 0.845	Z = 2.402	Z = 0.629
*P* value		0.301	0.396	**0.015**	0.533

**Table 3:** Correlation analysis between dependent variables and numerical confounding variables listed above

Parameter and group		Age	PMI	BW	BAC
CS, right AC	S *r/P*	− 0.05/0.83	0.11/0.62	− 0.12/0.59	− 0.38/0.07
	C *r/P*	− 0.37/0.08	− 0.05/0.81	− 0.11/0.59	0.10/0.65
CS, left AC	S *r/P*	− 0.13/0.56	0.05/0.82	− 0.12/0.57	− 0.11/0.62
	C *r*/*P*	− 0.18/0.41	− 0.12/0.59	− 0.17/0.43	− 0.07/0.75
LI	S *r/P*	− 0.10/0.63	− 0.26/0.21	0.18/0.40	0.36/0.08
	C *r*/*P*	0.38/0.07	− 0.52/**0.01**	− 0.18/0.41	− 0.14/0.52

q1 and q3 – quartile 1 and 3; n – number of cases; PMI – postmortem interval; BW – brain weight; BAC – blood alcohol concentration; S – suicide victims; C – controls; *r* – correlation coefficient and *P* – *P* value of the Spearman’s correlation. Significant *P* values are in bold

### Confounders

Suicidal and control groups were matched by sex (non-significant *χ*^*2*^-test *P* value, see Tables 1, 2, 3 and Supplementary Table). According to the effect of sex suggested by the initial GCD procedure, female subjects revealed higher CS activity in both groups bilaterally and the difference was significant in the left AC in controls (median values in females and males: 202.04 and 158.5 nmol × min^−1^ × mg^−1^ protein, respectively; *U*-test *P* = 0.036, corrected for multiple comparisons). However, very small numbers of female subjects in compared groups prevent from far-reaching conclusions regarding sex-specific differences in CS activity. Further analysis revealed that the laterality index was significantly increased only in male suicide victims compared to male controls (*U*-test *P* = 0.0003, corrected for multiple comparisons, see Tables 1, 2, 3). Therefore, the observed phenomenon of left-lateralised CS activity in the AC in suicide was specific for males.

Initial analyses by the GCD procedure revealed no associated impact of any of numerical confounders (i.e. age, PMI, BAC, and brain weight) and forensic diagnosis (i.e. suicides vs. controls) on both CS activity bilaterally and laterality index (non-significant Wald statistic *P* values). In the subsequent analysis by *U*-tests, age, PMI, and BAC revealed no significant differences between suicides and controls, whereas the brain weight was significantly higher in the former group (see Tables 1, 2, 3 and Supplementary Table). However, further Spearman’s correlations analysis did not suggest that either brain weight or other numerical confounders influenced the results of comparisons between CS activity or its laterality index in study groups (see Tables 1, 2, 3).

Moreover, no significant differences in investigated parameters were found between inebriated and remaining cases in the entire pool of results as well as in compared groups, also in hemisphere-specific statistical analyses (insignificant *H*-tests *P*-values followed by insignificant *U*-tests *P*-values corrected for multiple comparisons).

## Discussion

Our results suggest in suicide the left-lateralisation of CS activity in the AC, which plays a key role in behavioural regulation [30], profoundly disturbed in suicide victims [10, 16–18]. This effect was specific for male suicides, similar to our previous studies of prefrontal regions by morphological and molecular methods [17, 18]. However, the very small sample size of female subjects, which reflects disproportions observed in epidemiological studies of suicide [31], may lead to an underpowered analysis and there is a chance of false negative results. Despite of low female representation, our study suggests higher CS activity in females, which was also observed previously in postmortem studies of both human and animal brain as a presumable effect of estrogens ([25] and [32], respectively). Therefore, considering the effect of sex, our results should be replicated in larger cohorts with more numerous female samples. Other variables which may confound present results, among them postmortem interval, did not influence current results.

In the brain, glucose oxidative metabolism closely related to CS activity in the TCA cycle is fundamental for both glutamatergic and GABAergic neurotransmission [8]. Previous neuroimaging studies assessing glucose utilisation revealed consistently increased baseline glucose uptake in the left compared to the right AC in major depressive disorder (MDD) patients with the accentuation of this phenomenon related to depression severity (for a review see: [33]). Correspondingly, this lateralisation effect was also found in depressed suicide attempters [34]. A possible explanation of this phenomenon could be the augmented energetic demand in cellular components of the left compared to the right AC and the increased neurotransmission (predominantly glutamatergic). The TCA cycle with CS as a key enzyme is crucial for both energy supply from oxidative glucose metabolism and the replenishment of glutamatergic and GABAergic neurotransmitter pools [6–9] (Fig. 1). In accordance with neuroimaging data, the increased glucose utilisation seems to be a compensatory attempt in deteriorated cortical function. Hypothetically, the currently observed left-lateralised CS activity in the AC of suicide victims may also play a role in counteracting functional dysfunction. Alternatively, as the CS activity in the left AC in suicides and controls was almost similar, the observed effect could be related to the activity decrease (however, insignificant) of the right AC in the former compared to the latter group (see Tables 1, 2, 3 and Supplementary Table). Diminished function of the right AC was observed in some neuroimaging studies of depression [35] and suicidal behaviour [36]. The strongest negative correlation between the activity and depression severity scores was also found in this prefrontal region [37].

We cannot distinguish by our method, which cellular components of the AC are most involved in the observed CS activity lateralisation. Pyramidal neurons constitute roughly 30% of prefrontal cells, whereas other cellular populations in the PFC include inhibitory interneurons (10%), oligodendrocytes (45%), astrocytes (12%) and microglia (3%) [38, 39]. Therefore, oligodendrocytes constitute the most numerous cellular population in the PFC and the rate of glucose oxidation by the TCA cycle observed in cell cultures is almost as high in oligodendrocytes as in neurons [9, 40]. Moreover, the rate of citrate synthesis in astrocytes in cortical cell cultures is higher than that assayed in neurons [6]. Correspondingly, the effect currently observed in postmortem AC samples could be predominantly generated by glial cells.

On the other hand, however, neurons (mostly glutamatergic and only in small part GABAergic) account for approximately up to 80% of glucose oxidation and astrocytes contribute most of the rest during the baseline activity of the living brain [3, 7]. Besides the abnormalities of baseline glutamatergic activity in the PFC suggested in suicidal behaviour by in vivo functional studies [33, 34], the augmented GABAergic activity has been indicated by postmortem research on suicide of ours and others [41–43]. Therefore, the hypothetically increased activity of GABAergic neurons in the left compared to the right AC may also be involved in the effect observed in suicide victims in our current study.

Most probably, both neuronal and glial populations contribute to the observed left-lateralised CS activity in the AC of suicide victims. As we cannot resolve this question currently, a further molecular analysis of distinct cellular populations in the AC is needed to explain the relation between CS activity in neurons, astrocytes, and oligodendrocytes in the brain of suicide victims.

## Limitations

The present study has certain limitations that have to be considered: (1) a relatively small number of predominantly male cases was analyzed. Therefore, results have to be confirmed in a larger sample with more numerous female subjects. (2) The psychiatric diagnoses (also including substance use disorders) and the data on possible psychotropic medication preceding suicidal death were not available. However, our current study did not aim at analysis of relation between suicide and other mental disorders. Moreover, the most of experimental data suggest an upregulation of CS activity due to psychotropic medication. As we did not observe this effect in our cohort of suicide victims, the hypothetic impact of medication on our current results seems to be unlikely. (3) As we used bulk tissue homogenates, our method does not allow to differentiate between cell types which contribute to the observed phenomenon of left-lateralised CS activity in the AC of suicide completers.

## Conclusion

In summary, our results suggest a left-lateralised CS activity in the AC of male violent suicide victims. This may represent a presumable compensatory attempt, which could counteract prefrontal functional impairment. Alternatively, the observed lateralization of CS activity may also be related to lower activity of the right AC in the context of suicide. Our study results correspond with previous neuroimaging and postmortem data on deteriorated PFC function in depression and suicide. However, further research is needed for the insight into the observed phenomenon and its implications for the neurobiology of suicide.

## Supplementary Information

Below is the link to the electronic supplementary material.

Supplementary file1 (DOCX 22 KB)

## Data Availability

On behalf of all authors, the corresponding author states that the data being reported are accurate and are coming from the official source.
