# Supplementary material for: The left-lateralisation of citrate synthase activity in the anterior cingulate cortex of male violent suicide victims
Source: Eur Arch Psychiatry Clin Neurosci. 2022 Nov 9;273(6):1225–32. doi: 10.1007/s00406-022-01509-2 (PMC10449962; doi:10.1007/s00406-022-01509-2)
Supplement: Supplementary file 1 — Supplementary file1 (DOCX 22 KB) [file 406_2022_1509_MOESM1_ESM.docx]

**The left-lateralisation of citrate synthase activity in the anterior cingulate cortex of male violent suicide victims**

Karol Karnecki, Julian Świerczyński, Johann Steiner, Marta Krzyżanowska, Michał Kaliszan, Tomasz Gos

**European Archives of Psychiatry and Clinical Neuroscience**

Corresponding author:

Tomasz Gos, MD, PhD

Department of Forensic Medicine

Medical University of Gdańsk

ul. Dębowa 23

80-204 Gdańsk, Poland

E-mail: [gost@gumed.edu.pl](mailto:gost@gumed.edu.pl)

**Supplementary Table** Detailed diagnostic and demographic data, and the values of citrate synthase (CS) activity in the anterior cingulate cortex (AC) bilaterally, with laterality index (100×[left−right]/[left+right]) in suicide victims (n=24) and controls (n=24). *Other abbreviations:* PMI – postmortem interval; BW – brain weight; BAC – blood alcohol concentration; LOQ – limit of quantification (BAC = 0.2 g/l); q1 and q3 – quartile 1 and 3. Significant *P* values are in bold.

| Case ID | Cause of death | Sex | Age [yrs.] | PMI [hrs.] | BW [g] | BAC [g/l] | CS activity [nmol/min × mg protein^─1^]  AC left | CS activity  [nmol/min × mg protein^─1^]  AC right | Laterality index |
| --- | --- | --- | --- | --- | --- | --- | --- | --- | --- |
|  |  |  |  |  |  |  |  |  |  |
|  | SUICIDE VICTIMS |  |  |  |  |  |  |  |  |
| 1 | fall from building | m | 21 | 72 | 1495 | 2.05 | 202.34 | 151.66 | 14.32 |
| 2 | fall from building | m | 65 | 48 | 1360 | <LOQ | 185.49 | 163.16 | 6.40 |
| 3 | fall from building | m | 68 | 72 | 1315 | 1.09 | 148.93 | 134.77 | 4.99 |
| 4 | fall from building | m | 19 | 72 | 1570 | <LOQ | 166.59 | 160.53 | 1.85 |
| 5 | hanging | m | 54 | 24 | 1460 | 0.4 | 183.00 | 152.00 | 9.25 |
| 6 | hanging | m | 27 | 24 | 1520 | <LOQ | 180.00 | 160.00 | 5.88 |
| 7 | hanging | m | 50 | 24 | 1365 | 2.3 | 201.00 | 179.00 | 5.79 |
| 8 | hanging | m | 53 | 24 | 1480 | <LOQ | 212.00 | 187.00 | 6.27 |
| 9 | hanging | m | 47 | 24 | 1540 | 2.8 | 134.00 | 113.00 | 8.50 |
| 10 | hanging | m | 55 | 24 | 1370 | 1.8 | 146.00 | 144.00 | 0.69 |
| 11 | hanging | m | 20 | 120 | 1400 | <LOQ | 226.72 | 236.04 | -2.01 |
| 12 | hanging | m | 68 | 96 | 1565 | <LOQ | 130.92 | 104.76 | 11.10 |
| 13 | hanging | m | 34 | 48 | 1495 | <LOQ | 168.07 | 152.57 | 4.83 |
| 14 | hanging | m | 52 | 48 | 1510 | <LOQ | 200.45 | 204.47 | -0.99 |
| 15 | hanging | m | 25 | 96 | 1425 | 0.5 | 182.96 | 202.17 | -4.99 |
| 16 | hanging | m | 35 | 48 | 1405 | 0.24 | 175.18 | 158.00 | 5.16 |
| 17 | hanging | m | 54 | 96 | 1625 | <LOQ | 152.04 | 145.95 | 2.04 |
| 18 | hanging | m | 27 | 24 | 1380 | 1.37 | 128.36 | 128.34 | 0.01 |
| 19 | hanging | m | 20 | 24 | 1360 | 1.24 | 114.27 | 101.10 | 6.11 |
| 20 | hanging | m | 32 | 24 | 1670 | 0.69 | 148.09 | 131.09 | 6.09 |
| 21 | hanging | m | 71 | 48 | 1515 | <LOQ | 128.52 | 159.80 | -10.85 |
| 22 | hanging | f | 76 | 24 | 1200 | <LOQ | 197.00 | 183.00 | 3.68 |
| 23 | hanging | f | 73 | 24 | 1380 | <LOQ | 181.00 | 188.00 | -1.90 |
| 24 | hanging | f | 21 | 24 | 1530 | 1.9 | 287.00 | 204.00 | 16.90 |
| Suicide victims: ratio/median (q1, q3) | | 21m/3f | 48.5 (26, 60) | 36 (24, 72) | 1470 (1375, 1525) | 0.12 (0.00, 1.31) | 177.59 (147.05, 198.73) | 158.90 (139.39, 185.00) | 5.08 (0.35, 6.34) |
|  |  |  |  |  |  |  |  |  |  |
|  | CONTROLS |  |  |  |  |  |  |  |  |
| 25 | acute myocardial infarction | m | 52 | 120 | 1380 | <LOQ | 181.89 | 183.80 | -0.52 |
| 26 | acute myocardial infarction | f | 57 | 24 | 1230 | <LOQ | 190.00 | 183.00 | 1.88 |
| 27 | acute myocarditis | m | 54 | 24 | 1480 | 1.1 | 127.00 | 129.00 | -0.78 |
| 28 | acute pancreatitis | m | 40 | 72 | 1325 | <LOQ | 206.00 | 227.00 | -4.85 |
| 29 | sudden cardiac death | m | 50 | 24 | 1220 | <LOQ | 180.00 | 191.00 | -2.96 |
| 30 | sudden cardiac death | m | 52 | 24 | 1455 | 0.8 | 182.00 | 163.00 | 5.51 |
| 31 | sudden cardiac death | m | 57 | 24 | 1645 | <LOQ | 130.00 | 123.00 | 2.77 |
| 32 | sudden cardiac death | m | 36 | 24 | 1780 | <LOQ | 135.39 | 154.72 | -6.66 |
| 33 | sudden cardiac death | m | 67 | 48 | 1225 | <LOQ | 209.37 | 189.94 | 4.87 |
| 34 | sudden cardiac death | m | 58 | 48 | 1520 | <LOQ | 172.44 | 181.72 | -2.62 |
| 35 | sudden cardiac death | m | 49 | 72 | 1335 | <LOQ | 125.14 | 134.04 | -3.43 |
| 36 | sudden cardiac death | m | 64 | 72 | 1490 | <LOQ | 140.87 | 153.38 | -4.25 |
| 37 | haemorrhagic shock (gastrointestinal haemorrhage) | m | 57 | 72 | 1345 | 1.01 | 90.25 | 96.35 | -3.27 |
| 38 | cardiopulmonary arrest  (hepatic cirrhosis) | m | 61 | 24 | 1055 | 0.3 | 145.00 | 146.00 | -0.34 |
| 39 | cardiopulmonary arrest (infectious interstitial nephritis) | m | 50 | 96 | 1345 | <LOQ | 101.83 | 114.46 | -5.84 |
| 40 | cardiopulmonary arrest  (oropharyngeal neoplasm) | m | 63 | 96 | 1290 | <LOQ | 139.66 | 135.27 | 1.60 |
| 41 | pulmonary embolism | m | 65 | 168 | 1260 | 2.79 | 49.52 | 94.21 | -31.10 |
| 42 | acute respiratory failure (pulmonary tuberculosis) | f | 44 | 24 | 1155 | <LOQ | 179.00 | 176.00 | 0.85 |
| 43 | haemorrhagic shock (extrauterine pregnancy) | f | 22 | 96 | 1330 | <LOQ | 214.07 | 237.00 | -5.08 |
| 44 | sudden cardiac death | m | 37 | 24 | 1525 | 2.3 | 172.00 | 175.00 | -0.86 |
| 45 | traffic accident (car) | m | 24 | 72 | 1415 | 1.47 | 180.35 | 190.85 | -2.83 |
| 46 | traffic accident (car) | m | 26 | 96 | 1475 | 1.15 | 209.25 | 237.21 | -6.26 |
| 47 | traffic accident (car) | f | 55 | 24 | 1340 | <LOQ | 250.97 | 247.37 | 0.72 |
| 48 | transport accident (train) | m | 62 | 48 | 1378 | 2.8 | 215.53 | 218.84 | -0.76 |
| Controls: ratio/median (q1, q3) | | 20m/4f | 53 (42, 59.5) | 48 (24, 84) | 1345 (1275, 1478) | 0.00 (0.00, 1.06) | 175.72 (132.70, 198.00) | 175.50 (134.66, 190.93) | -1.74 (-4.55, 0.79) |
| Statistics |  |  |  |  |  |  |  |  |  |
|  | test | χ^2^-test | U | U | U | U | U | U | U |
|  | characteristic value | χ^2^ = 0.17 | Z = -1.041 | Z = -0.845 | Z = 2.402 | Z = 0.629 | Z = 0.577 | Z = -0.825 | Z = 3.763 |
|  | P value | 0.683 | 0.301 | 0.396 | **0.015** | 0.533 | 0.560 | 0.407 | **0.00009** |
